# Are rapid diagnostic tests more accurate in diagnosis of *plasmodium falciparum *malaria compared to microscopy at rural health centres?

**DOI:** 10.1186/1475-2875-9-349

**Published:** 2010-12-02

**Authors:** Vincent Batwala, Pascal Magnussen, Fred Nuwaha

**Affiliations:** 1 Department of Community Health, Mbarara University of Science & Technology P. O. Box 1410, Mbarara, Uganda; 2 Centre for Health Research and Development, Faculty of Life Sciences, Copenhagen University, Thorvaldsensvej 57, DK1871 Frederiksberg C, Denmark; 3 Disease Control and Environmental Health, Makerere University School of Public Health, P. O. Box 7072, Kampala, Uganda

## Abstract

**Background:**

Prompt, accurate diagnosis and treatment with artemisinin combination therapy remains vital to current malaria control. Blood film microscopy the current standard test for diagnosis of malaria has several limitations that necessitate field evaluation of alternative diagnostic methods especially in low income countries of sub-Saharan Africa where malaria is endemic.

**Methods:**

The accuracy of axillary temperature, health centre (HC) microscopy, expert microscopy and a HRP2-based rapid diagnostic test (Paracheck) was compared in predicting malaria infection using polymerase chain reaction (PCR) as the gold standard. Three hundred patients with a clinical suspicion of malaria based on fever and or history of fever from a low and high transmission setting in Uganda were consecutively enrolled and provided blood samples for all tests. Accuracy of each test was calculated overall with 95% confidence interval and then adjusted for age-groups and level of transmission intensity using a stratified analysis. The endpoints were: sensitivity, specificity, positive predictive value (PPV) and negative predictive value (NPV). This study is registered with Clinicaltrials.gov, NCT00565071.

**Results:**

Of the 300 patients, 88(29.3%) had fever, 56(18.7%) were positive by HC microscopy, 47(15.7%) by expert microscopy, 110(36.7%) by Paracheck and 89(29.7%) by PCR. The overall sensitivity >90% was only shown by Paracheck 91.0% [95%CI: 83.1-96.0]. The sensitivity of expert microscopy was 46%, similar to HC microscopy. The superior sensitivity of Paracheck compared to microscopy was maintained when data was stratified for transmission intensity and age. The overall specificity rates were: Paracheck 86.3% [95%CI: 80.9-90.6], HC microscopy 93.4% [95%CI: 89.1-96.3] and expert microscopy 97.2% [95%CI: 93.9-98.9]. The NPV >90% was shown by Paracheck 95.8% [95%CI: 91.9-98.2]. The overall PPV was <88% for all methods.

**Conclusion:**

The HRP2-based RDT has shown superior sensitivity compared to microscopy in diagnosis of malaria and may be more suitable for screening of malaria infection.

## Background

The World Health Organization (WHO) recommends prompt parasitological confirmation by microscopy or rapid diagnostic test (RDTs) for all patients with suspected malaria before treatment is started [1,2]. Treatment solely on the basis of clinical suspicion should be considered only when a parasitological and or RDT diagnosis is not accessible [1-3]. Parasitological based diagnosis of malaria is currently of global public health priority as use of more expensive and limited supply antimalarials increases [4-6], malaria infection and disease become rarer with increasing malaria control strategies [7] and for good clinical practice [3].

In most malaria endemic countries of sub-Saharan Africa, the current standard for laboratory confirmation of a clinical malaria diagnosis is a peripheral blood film, examined microscopically. However, microscopic based diagnosis of malaria is labour-intensive requiring trained staff and quality equipment attributes that are scarce in resource-poor settings [8,9]. Thus, most clinicians often rely on clinical signs and symptoms for diagnosis of malaria, even when slide microscopy is available [10,11]. Besides, when anti-malarials were relatively cheap, presumptive treatment of all fever cases was deemed more cost-effective [12].

Uganda piloted the histidine-rich protein II (HRP2)-based RDT (Paracheck) and rolled it out as an instrument of choice for parasite-based malaria diagnosis and patient management in six districts in the first phase in 2008. Paracheck was distributed to parish-level and to selected sub-county health centres (HCs) without laboratory infrastructure. RDTs have been out of stock for twelve months. Currently, there are 955 sub-county and 2,008 parish-level HCs in 112 districts in the country. Depending on availability of RDTs, scaling up to additional 22 districts is planned for January 2011.

Some data suggest that RDTs may not be very sensitive especially in varying transmission intensities [13,14]. However, most evaluations of RDTs have used expert microscopy as gold standard [15-17]. Since both microscopy and RDTs have limitations in identifying malaria infection [13,18,19], there is need to use a more accurate gold standard (such as PCR) in assessing the accuracies of these diagnostic tests. The aim of this study was to compare the diagnostic accuracy of a histidine-based RDT (Paracheck) to that of HC microscopy, expert microscopy and presumptive diagnostic method in diagnosis of malaria in rural HCs of Uganda, with PCR as gold standard.

## Methods

### Study setting

Data was collected from June to July 2007 in three randomly selected sub-county level government HCs in Bushenyi and three in Iganga districts of Uganda. Bushenyi is categorized as low and Iganga as high malaria transmission intensity settings. The annual entomological inoculation rates are not known, but are reported to be <10 and >500 infective bites per person per year in the neighboring districts of Kanungu and Tororo respectively [20]. The detailed description of the study sites has already been published [6].

A sub-county level HC laboratory is manned by one laboratory assistant who has two years of pre-service training. The laboratory personnel perform all investigations requested by the clinician. For malaria, at least one out of ten slides is stored daily for quality control. The district laboratory-focal person (who is also the in-charge of the district hospital laboratory) performs quarterly technical support supervision to HC laboratories. During supervision the microscope, stains, staining of slides and reporting are checked. The supervisor performs fresh films with the laboratory assistant. Where necessary, an immediate feedback is given, but also takes a percentage of the slides for further examination. The external quality assurance is coordinated at the national level. As contribution to improvement in the delivery of services, the study provided HC laboratories with new binocular microscopes, reagents and supplies.

### Sample size estimation

Using a nomogram [21], estimated malaria prevalence of 63.5% [22], *p*-value set at 0.05, with estimated sensitivity of Paracheck of 95% [23], a sample size of 150 in each district was appropriate totalling 300 patients for the two districts.

### Participants and eligibility

All male and female outpatients attending study centres with clinical suspicion of uncomplicated malaria based on fever and or history of fever within the previous 48 hours were eligible for inclusion in the study. Lack of consent and incomplete data constituted the exclusion criteria.

### Training of study team

The research team comprised of staff of the selected HCs (three clinical officers, three laboratory assistants, nine nurses) and three research assistants per district. A one-week residential training workshop was conducted in each district. Standard operating procedures for: 1) finger prick for collection of blood, 2) thick/thin blood smear preparation, 3) staining smears, 4) blood smear reading, 5) preparation and reading of Paracheck, and 6) collection of blood on filter paper for PCR were used in training. All members were trained in theory and practice during pilot testing of the patient case report forms (CRFs). Data collection commenced after inter-reader reliability for Paracheck reached a very good agreement (kappa coefficient = 0.97).

### Design and patient enrolment

**Figure 1:**
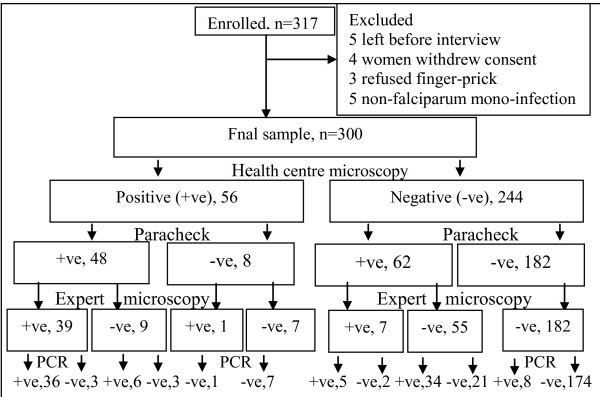
**Trial profile**.

This was a diagnostic clinical trial. The diagnosis of malaria was made in the outpatient department by the attending HC clinicians. Only those eligible were informed about the study. Those who gave consent were consecutively enrolled. Medical history, socio-economic and demographic data were recorded on CRFs. Figure 1 shows the trial profile.

### Description of laboratory procedures

#### Malaria microscopy

Blood for thick and thin smears, RDT and PCR were collected from the same finger-prick. Duplicate thick and thin smears were prepared on separate frosted slides bearing the patient's identification number. Standard staining was made using the "Field's stain" method. This method provides a readable film within few minutes compared to "Giemsa stain." The Field's stain was the only re-agent available in HCs and to which the laboratory assistants were familiar. Blood slides were read at the HCs (magnification x1,000) under natural light (none of the study HCs had electricity). Each film was graded as positive (asexual malaria parasites seen) or negative (no malaria parasites seen) based on the inspection of 200 fields of the thick smear. The parasite density was estimated assuming 8,000 white blood cells/μl [24]. The thin smear was useful in species identification. The laboratory assistants were blinded of the RDT results. All slides were stored in secured slide-boxes and read by an expert microscopist at Mbarara Regional Referral/University Teaching Hospital located in western Uganda. For quality control, all slides were re-read by another expert microscopist at Makerere University School of Public Health in Kampala. The expert microscopist was blind to HC microscopy and RDT results. The readings of the expert microscopist and that of the quality control microscopist were discrepant in seven slides. In these cases, the judgement of another senior laboratory technician was final.

All HC laboratory personnel had ≥4 years of work experience at the study HCs. The expert microscopist was a senior laboratory technician with eight years of work/research experience at Mbarara Regional Referral/University teaching hospital. Quality control was performed by senior laboratory personnel with over nine years of work/research experience.

#### Paracheck Pf

The choice of Paracheck Pf device (Orchid Biomedical Systems, Goa, India) was based on its stability [3], low cost, reported high sensitivity (97%) and moderate specificity (88%) in controlled trials, ease of use [16] and it was likely to be preferred by the Uganda Ministry of Health. About 5μl of blood was drawn by the laboratory assistant using a loop provided with the device. The test preparation and interpretation was done following manufacturer's instructions. Each test was read by two trained nurses. The nurses were not aware of the microscopy results. The test was considered positive when the antigen and control lines were visible in their respective windows, negative when only the control band was visible and invalid when the control band was not visible. Faint test lines were considered positive. The readings were discrepant in two faint test lines, where the judgement of a third trained nurse was final.

#### The PCR assay

The *Plasmodium falciparum *species-specific nested PCR was preferred because this species contributes majority of malaria morbidity in Uganda. Those infected with *P. falciparum *are likely to suffer poor outcomes if they are not appropriately managed. Finger-prick blood was blotted on Whatman 3 MM filter paper, dried in dust free area, wrapped inplastic sample bags and placed in a zip-lock bag with silica gel to prevent DNA degradation. The samples were sent to Makerere University-University of California San Francisco Molecular Research Laboratory http://muucsf.org/index.html located in Mulago National Referral Hospital in Kampala for analysis. DNA was extracted from filter paper using the chelex method [25]. *Plasmodium *genus-specific PCR, followed by *P. falciparum *species-specific nested PCR of 18 S small sub-unit ribosomal DNA was performed following a standard protocol [26]. PCR products were stained by ethidium bromide and resolved by gel electrophoresis on a 2.5% agarose gel. DNA size standards were separated alongside PCR products to allow sizing of species bands. Upon completion of the gel electrophoresis, gels were placed in a gel imaging cabinet and digitally photographed under ultraviolet light. Gel images were printed and corresponding sample lanes scored visually for the presence of *P. falciparum*. Positive and negative controls were used for each round of PCR. In addition, twenty two samples were randomly selected plus eight samples that were positive by PCR only (10% of the total) for re-analysis as a quality control measure. Quality assurance is performed by the University of California San Francisco, USA.

### Patient management

The field microscopy results and that of Paracheck were forwarded to the clinician to guide on the treatment decision. All patients with positive test results (slide or Paracheck) were immediately treated with artemether-lumefantrine (the current first-line anti-malarial) on the same day of visit. Patients with negative results received further assessment and an appropriate treatment strategy was given.

### Statistical analysis

Data was double-entered and validated in EpiData version 3.1 software (The EpiData Association, Odense, Denmark) and analysed using Stata version 10 (Stata Corp, Lakeway, College Station, Tx, USA). The sensitivity, specificity, PPV and NPV of HC microscopy, expert microscopy, axillary temperature diagnosis and RDT were determined with PCR as gold standard. A sub-analysis with expert microscopy as gold standard was performed to compare the results with that of PCR. Sensitivity was calculated as the proportion of positive test results obtained among samples containing malaria parasites by PCR, specificity as the proportion of negative test results obtained among samples whose PCR results were negative. PPVs and NPVs were calculated as the proportion of true-positive results among all positively reacting samples and as the proportion of true negative results among all negatively reacting samples, respectively. Accuracy of each test was calculated overall with 95% confidence interval (CI) and then adjusted for age-groups and level of transmission intensity using a stratified analysis. Because PCR was *P. falciparum *species-specific, and yet Paracheck only detects *P. falciparum*, five non-falciparum mono-infections (three *Plasmodium malariae *and two *Plasmodium ovale*) were excluded in the analysis.

### Ethical approval

The study was approved by Makerere University School of Public Health Institutional Review Board; and the Uganda National Council for Science and Technology (Ref: HS 209). Written informed consent was sought from participants (or parents/legal guardians for minors) at the time of interview. The study is registered with the Clinicaltrials.gov (NCT00565071).

## Results

### Socio-demographic profile of subjects

Three hundred seventeen patients were enrolled from June to July 2007. Five patients left before interview, four female patients withdrew consent because they wanted to consult their husbands, three feared the finger-prick and it was not possible to get blood specimens while five had non-*falciparum *mono-infection. The final sample was 300 patients. All were rural and majority were peasants. Their mean age was 17.1 years (range three months to 88 years). Those under five years of age were 117 (39.1%) while 175 (58.5%) were below 14 years. Females constituted 191(63.7%) of the sample. One hundred thirty four patients (44.7%) slept under insecticide treated mosquito net a night prior to enrolment into the study (Table 1).

**Table 1:** Selected characteristics of study participants

Selected variable	Overall n(%)	Low transmission n(%)	High transmission n(%)	*p*-value (2-sided)
Proportion <5 years of age	117(39.1)	52(34.7)	65(43.3)	0.124
Sleeps under mosquito net	134(44.7)	78(52)	56(37.3)	0.015
Used anti-malarial prior to visiting study health centre	58(34.1)	20(20.6)	38(52.1)	0.000
Axillary temperature ≥37.5°C	88(29.3)	40(26.7)	48(32.0)	0.375
Health centre microscopy slide positive	56(18.7)	11(7.3)	45(30.0)	0.000
Expert microscopy slide positive	47(15.7)	5(3.3)	42(28.0)	0.000
Paracheck positive	110(36.7)	22(14.7)	88(58.7)	0.000
PCR positive	89(29.7)	12(8.0)	77(51.3)	0.000

PCR = Polymerase Chain Reaction

The mean duration of illness before reporting to study HCs was 4.0 days [95%CI: 3.3-4.7] for children under-five years and 5.9 days [95%CI: 4.5-7.4] for those ≥5 years. At least 170(56.6%) had used some medications before reporting to study HCs. These included antimalarials 58(34%), analgesics 125(73.5%) and antibiotics 43(25.3%). Some patients used combinations of medicines.

### Overall results of diagnostic techniques

Out of 300 patients, 88(29.3%) had fever (temperature ≥37.5°C) with a mean of 38.3°C. Fifty-six (18.7%) slides were positive by HC microscopy and 47(15.7%) by expert microscopy (Table 1). The geometric mean of asexual parasitaemia was 111/μl. Paracheck detected 110 (36.7%) positive cases and 89 (29.7%) by PCR. The PCR gave positive results in eight patients who were negative with microscopy and Paracheck. Out of 58 patients who had used anti-malarials, the following tested positive: HC microscopy 16 (27.6%), expert microscopy 13 (22.4%), Paracheck 27 (46.6%) and PCR 26 (44.8%). Their geometric mean of asexual parasitaemia was 96.4/μl.

### Sensitivity and specificity of diagnostic techniques

Basing on PCR as gold standard (Table 2) the overall sensitivity of presumptive diagnosis based on axillary temperature, HC microscopy, expert microscopy and Paracheck were: 39.3%, 47.2%, 46.1% and 91% respectively. The corresponding specificity rates were: 74.9%, 93.4%, 97.2% and 86.3% respectively. With a sub-analysis using expert microscopy as gold standard, the overall sensitivity for presumptive diagnosis based on axillary temperature, HC microscopy and Paracheck were: 42.6%, 85.1% and 97.9% respectively. The corresponding specificity rates were: 73.1%, 93.7% and 74.7% respectively.

**Table 2:** Overall sensitivity, specificity, PPV and NPV of malaria diagnostic methods with PCR as gold standard

Diagnostic techniques	Sensitivity %(number/total) [95%CI]	Specificity %(number/total) [95%CI]	PPV %(number/total) [95%CI]	NPV %(number/total) [95%CI]
Axillary temperature ≥37.5°C	39.3(35/89) [29.1-50.3]	74.9(158/211) [68.5-80.6]	39.8(35/88) [29.5-50.8]	74.5(158/211) [68.1-80.2]
Health centremicroscopy	47.2(42/89) [36.5-58.1]	93.4(197/211) [89.1-96.3]	75.0(42/56) [61.6-85.6]	80.7(197/244) [75.2-85.5]
Expert microscopy	46.1(41/89) [35.4-57.0]	97.2(205/211) [93.9-98.9]	87.2(41/47) [74.3-95.2]	81.0(205/253) [75.6-85.7]
Paracheck	91.0(81/89) [83.1-96.0]	86.3(182/211) [80.9-90.6]	73.6(81/110) [64.4-81.6]	95.8(182/190) [91.9-98.2]

PPV = Positive Predictive Value, NPV = Negative Predictive Value, 95%CI = 95% Confidence Interval

In the low transmission setting, sensitivity of Paracheck was 75%. The sensitivity of presumptive diagnosis based on axillary temperature, HC- and expert microscopy was very low (25% for each). Unlike HC microscopy 94.2% [95%CI: 88.9-97.5], the specificity of expert microscopy 98.6% [95%CI: 94.9-99.8] was significantly higher than that of Paracheck 90.6% [95%CI: 84.4-94.5], *p *= 0.004.

In the high transmission setting, the sensitivity of Paracheck was 93.5% [95%CI: 85.5-97.9] and significantly higher than that of other diagnostic methods (*p *< 0.001 for each comparison). Its specificity (78.1%) was significantly lower than that of HC microscopy 91.8% [95%CI: 83-96.9] (*p *< 0.001) or expert microscopy 94.5% [95%CI: 86.6-98.5] (*p *< 0.001), but similar to presumptive diagnosis based axillary temperature.

With regard to age, the sensitivity of Paracheck was significantly higher than that of other techniques and was excellent in children <5 years of age 97.7% [95%CI: 88-99.9] compared to those ≥5 years 83.7% [95%CI: 69.3-93.2]. The specificity of Paracheck in children <5 years was 79.5% [95%CI: 68.4-88.0] while it was 89.9% [95%CI: 83.6-94.3] in those ≥5 years. The specificity of HC microscopy 95.7% [95%CI: 90.8-98.4] in patients ≥5 years was not different from that of expert microscopy 98.6% [95%CI: 94.9-99.8]. In addition, the specificity of HC microscopy 89.0% [95%CI: 79.5-95.1] in children <5 years was not statistically different from that of expert microscopy 94.5% [95%CI: 86.6-98.5].

### Positive and negative predictive values

Overall, only Paracheck had a NPV of >90%, while the PPVs for all methods were <88%. In the low transmission setting, PPV was low for all diagnostic methods: axillary temperature (7.5%), HC microscopy (27.3%), expert microscopy (60%) and Paracheck (40.9%). In the high transmission areas, the PPV for presumptive diagnosis (66.7%) was significantly lower than for other diagnostic methods. In addition, the PPV for expert microscopy 90.5% [95%CI: 77.4-97.3], HC microscopy 86.7% [95%CI: 73.2-94.9] and Paracheck 81.8% [95%CI: 72.2-89.2] were statistically not different. The NPV for Paracheck 97.7% [95%CI: 93.3-99.5] in low transmission and 91.9% [95%CI: 82.3-97.3] in high transmission were significantly higher than that of other methods. Only Paracheck had a NPV >90% in both age-groups, being 94.7% [95%CI: 89.3-97.8] in patients ≥5 years and 98.3% [95%CI: 90.9-100] in those <5 years.

## Discussion

The accuracy of clinical diagnosis routinely practiced at rural HCs, health centre microscopy, expert microscopy and Paracheck was compared in patients with uncomplicated malaria. The diagnostic accuracy of these methods was measured against PCR as gold standard. Some studies have however reported on the accuracy of RDTs using expert microscopy as gold standard [15-17]. For a balanced comparison, a sub-analysis using expert microscopy as gold standard was also performed.

If expert microscopy was the gold standard, the overall sensitivity was consistently high. The sensitivity (97.9%), specificity (74.7%) and NPV (99.5%) of Paracheck were similar to that reported elsewhere [13,16,17,27]. When PCR was used as gold standard, the sensitivity of Paracheck was 91%, specificity 86.4% and NPV 95.8%. The sensitivity of expert microscopy (46.1%) was unacceptably low and similar to that of HC microscopy. A number of factors might have contributed to the low sensitivity of microscopy including the inherent limitations of microscopy [19], existence of low density infections and inappropriate use of anti-malarials [28] resulting into low parasitaemia. The low sensitivity of microscopy demonstrated here is an eye-opener to yet another limitation not only to the use of malaria microscopy as gold standard in research but also to interpretation of results in routine patient care. Indeed this finding substantiates the clinicians' concerns that influence them specifically not to adhere to negative malaria microscopy results [10,11]. Although low parasitaemic patients are less at risk from severe clinical malaria episodes, they perpetuate parasite transmission, and are still a public health concern [23]. For confident diagnosis of malaria in a routine outpatient practice, a sensitivity of >90% is critical [29] and this was only achieved by Paracheck.

This study reports that 37% and 47% patients who were negative by HC microscopy and expert microscopy respectively were confirmed to have malaria by PCR. These rates are slightly lower than that of another study [12], which reported that 67% of patients, who had been classified as negative by expert microscopy, were actually positive by PCR. On the other hand, the rapid test identified positive cases in excess of the gold standard, likely to be patients with persistently circulating antigen due to prior use of anti-malarials. Microscopy techniques fell short of the required critical level of sensitivity with the potential consequences of missing infections in individuals who might even have had low immunity. Paracheck detected majority of malaria cases but also led to treatment of a small percentage of patients without malaria infection.

Eight patients were negative with microscopy and Paracheck, but positive with PCR. Even a repeat of the analysis during quality control, the eight samples remained positive. An earlier investigation [30] into the disappearance of *P. falciparum *during treatment found that PCR remained positive for a median of 144 hours. In another study [31] PCR detected *P. falciparum *DNA from circulating nonviable parasites after successful treatment. However, HRP2-based RDTs remain positive after treatment, and the HRP2 signal is of no value during the first week of treatment [32]. Therefore, the eight patients with positive PCR and negative HRP2-based RDT reported here may represent *P. falciparum *with an HRP2 gene deletion or reduced HRP2 expression [18], and such patients never give a positive result with these tests [33].

The overall specificity of Paracheck was lower than that of HC- and expert microscopy. This pattern was also shown when data was adjusted by transmission intensity and age-groups. The low specificity rates have been attributed to persistent antigenaemia even after successful treatment in some reports [18,23,27,34], which is an inherent weakness in HRP2-based tests. In the current study however, out of the 27 patients who had prior use of antimalarials and were positive by Paracheck, only one was declared negative by PCR. It is likely that although they had used anti-malarials, they were still infected with malaria parasites.

Uganda has adopted RDT as a method for parasitological diagnosis of malaria in addition to microscopy [35]. RDTs are rolled out in lower level HCs where microscopy services are not functional or not available. The low sensitivity of HC microscopy reported here is an indicator that the quality of malaria case diagnosis greatly needs to be improved. This might involve strengthening HCs through in-service training, being equipped with adequate malaria diagnostic supplies, improved technical laboratory support supervision, and external quality assurance. Due to patient load (one microscope serving 25,000 people at sub-county level HC) and with laboratory investigations other than malaria being requested, 20-30 malaria slides can be examined satisfactorily per day. Therefore, many patients are likely to be treated presumptively. If a steady supply of RDTs is guaranteed, the distribution should be extended to all lower level facilities. In addition, it is vital to routinely evaluate the performance of RDTs as they are being rolled out in the country. Although microscopy has limitations [19,36] plus the low sensitivity reported here, it is useful in estimating the level of parasitaemia in a blood film as well as for detecting non-*falciparum *infections [35].

The PCR assay was used as the gold standard because it has the ability to detect malaria parasites in patients with low levels of parasitaemia. Infections with ≤5 parasites per μl can be detected with 100% sensitivity and equal specificity [26]. However, PCR also has limitations. PCR might give false negative results if samples containing the parasites fail to amplify because the target sequence recognized by oligonucleotide primers is absent or because it is present but inaccessible. Absence of the target sequence may be due to deletion/mutation of sequence homologous to the primers, degradation of DNA during sample preparation and storage. Alternatively, if the correct target sequence is present, amplification may fail due to inhibition of PCR by sample components. Also, target DNA may not be accessible because of inadequate cellular lysis, or the target sequence copy number may be too low for amplicons to be detected under conditions used. False positive PCR results might arise from carry over during sample processing [37]. The urgency and importance of obtaining results quickly for patients with suspected malaria limits the usefulness of PCR in routine clinical practice. Furthermore, in malaria endemic areas, limited financial resources, persistent sub-clinical parasitaemia and inadequate laboratory infrastructures in remote settings preclude PCR as a diagnostic method. Nonetheless, PCR remains a reference tool both clinically and in research [38].

In estimation of the sample size with the aid of nomogram [21], the prevalence of 63.5% was used. This study reports the overall prevalence to be 36.7%. With all other assumptions remaining constant, and using a nomogram, the prevalence of 63.5% gives the same sample size as 36.7%. Therefore, the power of the study was not affected.

## Conclusion

High sensitivity of malaria diagnosis is important in all settings, and essential for the most vulnerable population groups in which malaria infection produces an acute illness that can rapidly progress to death. The HRP2-based test demonstrated a superior sensitivity compared to microscopy and presumptive methods in the diagnosis of uncomplicated malaria in remote health facilities. Based on the current findings, the HRP2-baed RDT may be more suitable for screening of malaria infection in routine practice in primary health care centres.

## Conflicts of interests

The authors declare that they have no competing interests.

## Authors' contributions

All authors conceived and designed the study; VB and FN collected, analysed, interpreted the data and drafted the manuscript; PM critically revised the manuscript. All authors read and approved the final manuscript.
